# Electric-field-driven magnetic domain wall as a microscale magneto-optical shutter

**DOI:** 10.1038/s41598-017-00365-8

**Published:** 2017-03-21

**Authors:** Nikolai E. Khokhlov, Anastasiya E. Khramova, Elena P. Nikolaeva, Tatyana B. Kosykh, Alexey V. Nikolaev, Anatoly K. Zvezdin, Alexander P. Pyatakov, Vladimir I. Belotelov

**Affiliations:** 1 Faculty of Physics, M.V. Lomonosov Moscow State University, Moscow, 119991 Russia; 2 Russian Quantum Center, Skolkovo, Moscow 143025 Russia; 3 Moscow Institute of Physics and Technology (State University), Dolgoprudny, 141701 Russia

## Abstract

Nowadays, spintronics considers magnetic domain walls as a kind of nanodeviсe that demands for switching much less energy in comparison to homogeneous process. We propose and demonstrate a new concept for the light control via electric field applied locally to a magnetic domain wall playing the role of nanodevice. In detail, we charged a 15-μm-thick metallic tip to generate strong non-uniform electric field in the vicinity of the domain wall in the iron garnet film. The electric field influences the domain wall due to flexomagnetoelectric effect and causes the domain wall shift. The resulting displacement of the domain wall is up to 1/3 of domain width and allows to demonstrate a novel type of the electrically controlled magneto-optical shutter. Polarized laser beam focused on the electric-field-driven domain wall was used to demonstrate the concept of a microscale Faraday modulator. We obtained different regimes of the light modulation – linear, nonlinear and tri-stable – for the same domain wall with corresponding controllable displacement features. Such variability to control of domain wall’s displacement with spatial scale of about 10 μm makes the proposed concept very promising for nanophotonics and spintronics.

## Introduction

Nowadays, local manipulation of the magnetization is of prime research interest in spintronics, quantum information and nanophotonics. Thus it has been demonstrated that magnetostatic spin waves in magnetic dielectrics are promising candidates for quantum data processing and storing in the form of qubits^1, 2^. However, for reading and writing of the spin wave based qubits one has to control spins at submicron scale. Local manipulation of the magnetic moments is strongly required in spin electronics as well^3^. In this view utilizing a domain wall (DW) may be the solution for various challenges in spintronics since the DWs may be considered as nanometer-scaled elements of magnonic nanocircuitry: channels for the control of spin-wave propagation^4^, deflectors^5^ (even with negative refraction^6^), tunable sources of short-wavelength spin waves^7^ and many others. Similar approach to the DW as a nano-sized device may be used in magneto-photonics aimed towards miniature magneto-optical devices^8–10^. At the same time, to seize this opportunity, one should be able to apply local influence at several microscale.

Conventional approach of magnetic field generation by inductive elements does not allow necessary level of locality. Apart from that, inevitably increased response time and Joule heat are other crucial obstacles for the applications. Local and ultrafast control of the magnetization is possible by femtosecond laser pulses due to the opto-magnetic and photo-magnetic effects^11–14^. But using the technique for DW manipulation is rather challenging: it has been experimentally demonstrated for the first time in 2016 only^15^. On the other hand, the emerging trend for low energy consumption spintronics relates to the magnetoelectric materials^16–18^, enabling implementation of the so called ‘gating’ technique, i.e. electric field driven modulation of magnetic state due to the intrinsic coupling of magnetic and electric subsystems.

The electric field modulation of the magneto-optical Faraday rotation is called electro-magnetooptical effect. It was first observed in the yttrium iron garnet (YIG) samples sandwiched between two transparent plane electrodes^19^. However even for the 100 µm-thick YIG plates the electric field induced variation of the Faraday angle was about several seconds of arc that was too small for practical application.

In our recent experiments (reviewed in ref. 20) we showed that application of electric field by the electrically charged tip (forming the gate) provides displacement of the magnetic DW. Such approach is promising to overcome the mentioned difficulties as there is no currents flow and some expensive devices with metallic tip or electrode approach. The origin of electric-field-driven DW motion is inhomogeneous magnetoelectric^21^ or flexomagnetoelectric^22^ interaction that gives rise to an electric polarization associated with DW. First indirect observation of this local ferroelectricity was done as DW motion in electric field of charged tip^23^ and has recently been detected directly by single molecule spectroscopy technique^24^.

Since electrically controlled DW between two adjacent domains can be treated as a shutter influencing polarized light, the problem of the electro-magneto-optical light control can be revised from the new perspective. This paper reports on the first experimental implementation of the electrically driven DW as nanodevice for magneto-optics: we treated DW in the bismuth substituted iron-garnet magnetic film as a nanoshutter and displaced it via electric field to modulate transmitted light at microscale. The approach provides the superior locality of the manipulation of the spin texture without electric current flow, some inductive elements, and expensive equipment.

## Results

### Domain wall, tip and laser spot relative positions

**Figure 1:**
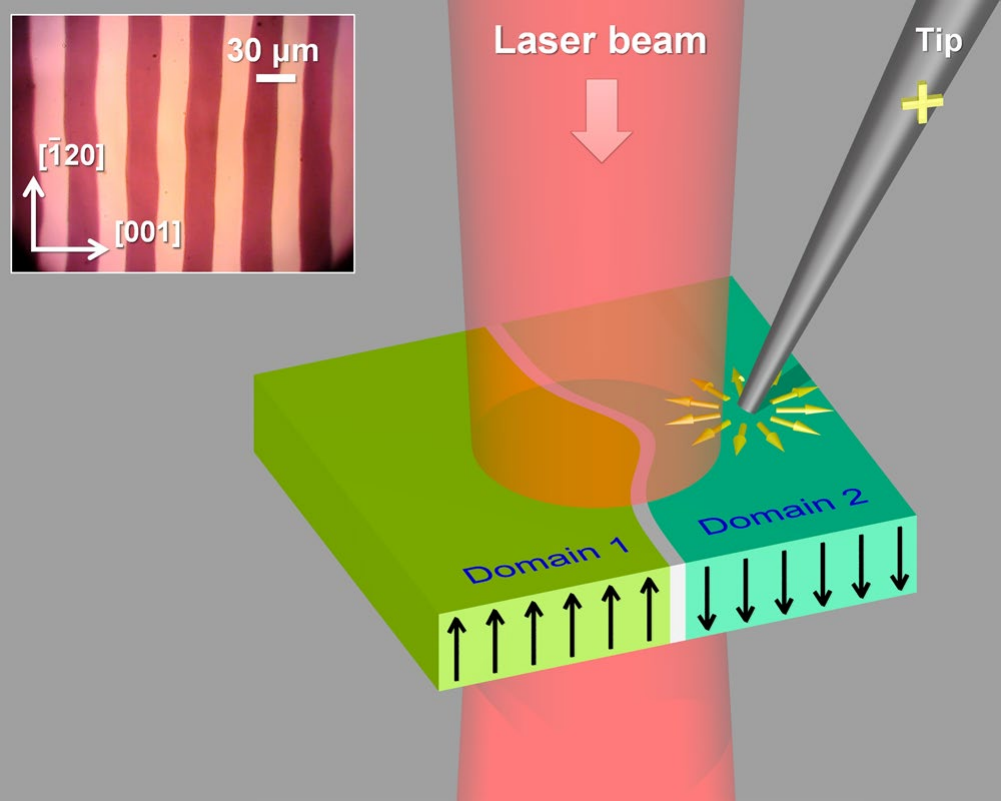
General layout of the experiment and stripe domains structure of the sample. Yellow arrows show schematically the electric field of the tip; black arrows – magnetizations of the adjacent domains; bright region between the domains is domain wall. The flexomagnetoelectric effect causes domain wall shift resulting in bulge shape of the wall. (Inset) Magneto-optical image of the stripe domain structure of the sample – 7.4-μm-thick epitaxial iron garnet film of composition (BiLu)_3_(FeGa)_5_O_12_ – obtained with the polarization microscope. Angle between the polarizer and analyzer is 20°; external magnetic field of 8 mT is in-plain and perpendicular to the DWs.

The experimental studies were performed with diode laser (wavelength λ = 660 nm) and 7.4-µm thick (BiLu)_3_(FeGa)_5_O_12_ film grown on (210) gadolinium gallium garnet substrate by liquid phase epitaxy. The film demonstrates specific Faraday rotation of 0.61°/μm at λ = 660 nm and has a stripe domain pattern (Fig. 1, inset).

Figure 1 illustrates the general layout of the experiment: strongly non-uniform local electric field is generated in the vicinity of the DW by applying a voltage between a metal tip and sample holder. With the electric voltage applied to the tip the flexomagnetoelectric effect takes place and the DW is shifted towards to or away from the tip (the cases of attraction and repulsion, respectively) depending on the DW chirality and the sign of the tip’s voltage^25^.

The origin of the electric-field-driven DW motion is inhomogeneous magnetoelectric^21^ or flexomagnetoelectric^22^ interaction characterized by the thermodynamic potential^26, 27^:

1 $${\rm{\Phi }}({\bf{P}},{\bf{M}})=\frac{{{\bf{P}}}^{2}}{2{\chi }_{e}}+\gamma {\bf{P}}\cdot [{\bf{M}}(\nabla \cdot {\bf{M}})-({\bf{M}}\cdot \nabla ){\bf{M}}+\ldots ],$$

where **M** is the magnetization vector, **P** is the electric polarization of DW, *χ*_*e*_ is the dielectric susceptibility, *γ* is the flexomagnetoelectric coupling constant; omitted terms do not contribute to the uniform polarization. In accordance to equation (1) the inhomogeneous flexomagnetoelectric interaction provides electric dipole moment of DW:

2 $${\bf{P}}=\gamma {\chi }_{e}[({\bf{M}}\cdot {\rm{\nabla }}){\bf{M}}-{\bf{M}}({\rm{\nabla }}\cdot {\bf{M}})].$$

According to equation (2), Néel walls with in-plane rotation of the magnetization vector can be the source of a spontaneous non-uniform electric polarization, while Bloch walls are electrically uncharged^20, 24, 27, 28^. To increase **P** we applied to the iron-garnet sample external magnetic fields: in-plane and normally to the sample surface (see Fig. S1 in Supplementary 1). The in-plane magnetic field tunes an internal magnetic structure of the DW closer to Néel-type and, thus, increases the DW displacement^20^. The out-of-plane field is applied to equalize the domain widths in the in-plane field.

**Figure 2:**
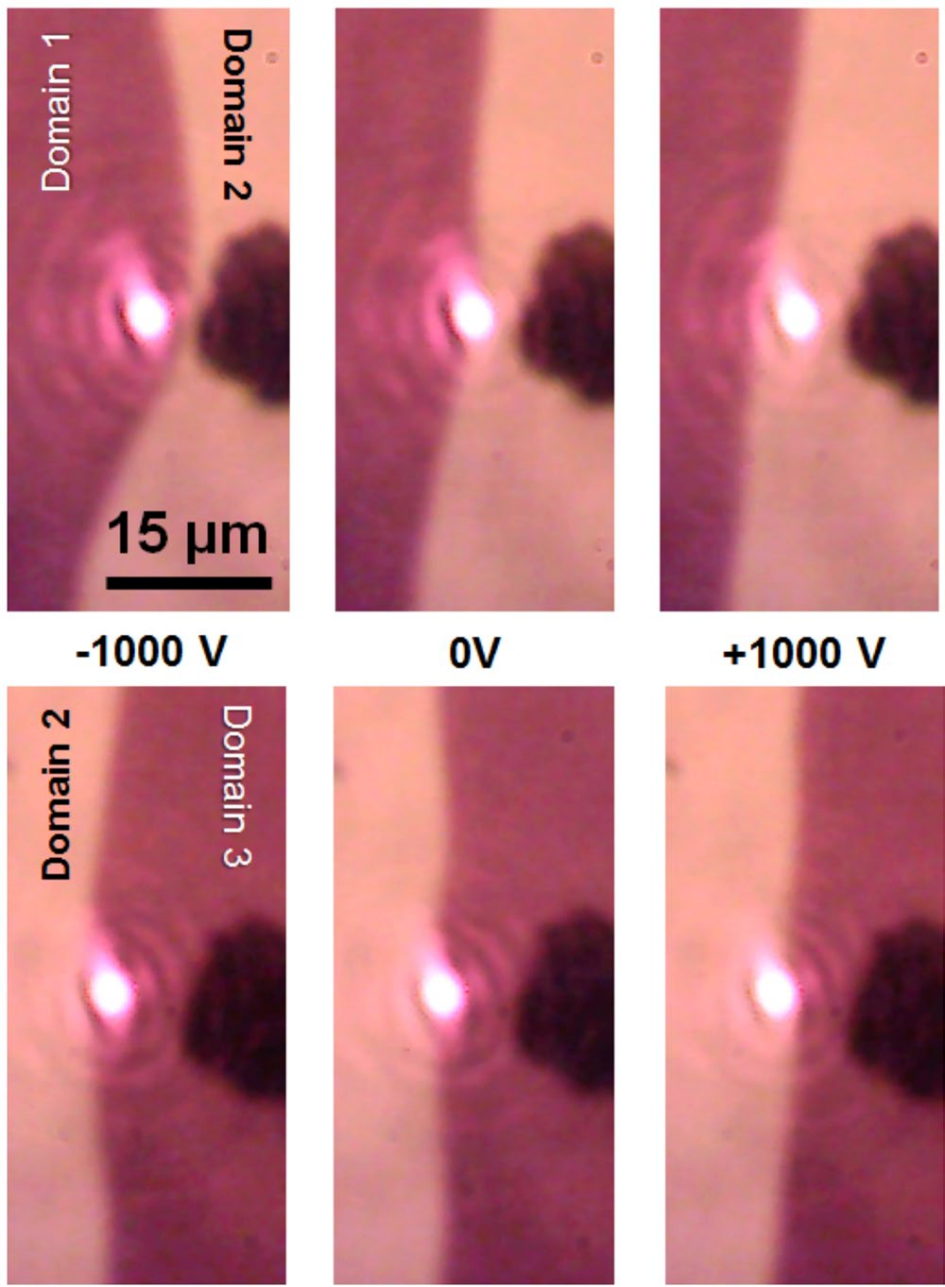
Magneto-optical image of the domain structure in the presence of local electric field. The domain wall is shifted from the equilibrium position due to the flexomagnetoelectric effect when the tip is charged. Dark and bright colors correspond to the opposite magnetization directions of the domains. Top and bottom rows demonstrate deformation of the left and right DWs of the same domain (the brighter one, domain 2). The tip voltages are −1000 V, 0 V, and +1000 V. The external in-plane magnetic field is 12 mT and is directed perpendicular to the DW. Out-of-plane field is about 0.15 mT. Bright spot in the middle is laser spot of 2 μm in diameter; laser wavelength is 660 nm.

The shifted DW strives to the linear state due to the magnetostatic restoring force proportional to the DW displacement^29^. The proportionality factor is determined by the saturation magnetization of the sample and gradient of effective field **H**_eff_. **H**_eff_ is the combination of three fields acting on DW: bias field, demagnetization field, and effective field of surface tension of the curved DW. DW is in equilibrium if **H**_eff_ = 0 with corresponding zero restoring force. Non-zero tip’s electric field shifts the DW and the restoring force rises. As a result the DW has bulge-like shape clearly seen in Fig. 2 and resembles a guitar string pulled away. DW goes back to the equilibrium line shape due to the restoring force when the voltage is off.

To obtain maximal possible strength of DW - charged tip interaction we had to bring the tip as close as possible to the DW. However, the minimum tip-to-DW distance is limited since it is necessary to avoid light blockage by the tip. As a result, the measurements of the laser intensity modulation were performed for the fixed initial tip-to-DW distance.

The level and the character of the light modulation are determined not only by the DW displacement but also by the angle *β* between the polarizer and the analyzer as well as by the initial positioning of the laser spot with respect to the DW. Generally, when the tip’s voltage is off the laser spot covers areas of two adjacent domains in proportion *a* to (1 − *a*). These two illuminated areas provide the Faraday angle of +*φ* and −*φ*, respectively. The intensity of the light transmitted through the system “polarizer – two domains - analyzer” is given by

3 $${I}_{0}=\zeta [a({\rm{1}}-p){\cos }^{{\rm{2}}}\,(\beta -\varphi )+(1-a)(1-p){\cos }^{{\rm{2}}}\,(\beta +\varphi )+p],$$

where *p* takes into account polarization non-ideality of the real experiment, *ζ* is a coefficient for absorption and scattering losses^30^. When the tip’s electric field is switched on the DW displacement modifies the ratio of the illuminated domain areas to *b* and (1 − *b*). It leads to the intensity modulation depth *δ*:

4 $$\delta =\frac{{I}_{e}-{I}_{0}}{{I}_{0}}=\frac{(b-a)\sin \,({\rm{2}}\beta )\sin \,({\rm{2}}\varphi )}{a{\cos }^{{\rm{2}}}\,(\beta -\varphi )-({\rm{1}}-a){\cos }^{{\rm{2}}}\,(\beta +\varphi )+p/(1-p)},$$

where *I*_*e*_ and *I*_*0*_ are transmitted light intensities when the tip’s voltage is switched on and off, respectively. Here we worked in the regime of *β* = 45°. The rise of *β* brings higher values of *δ* up to several hundred of percent (limited by the term *p*/(1 − *p*) in denominator of equation (4)) but with the decrease of signal-to-noise ratio.

With respect to the “traditional” magneto-optical modulator with magnetization driven uniformly in the entire film, here we have one more degree of freedom represented by the parameter *a*. For *β* = 45° the optimal case is *a* = 0.5 as it maximizes the derivative *∂δ*/*∂a* in equation (4). Notably, one could also choose the sign of modulation since adjacent domains have opposite magnetizations. Therefore, if in the attraction regime some DW provides positive change of light intensity then the next one gives negative change and vice versa.

### Modulation regimes

**Figure 3:**
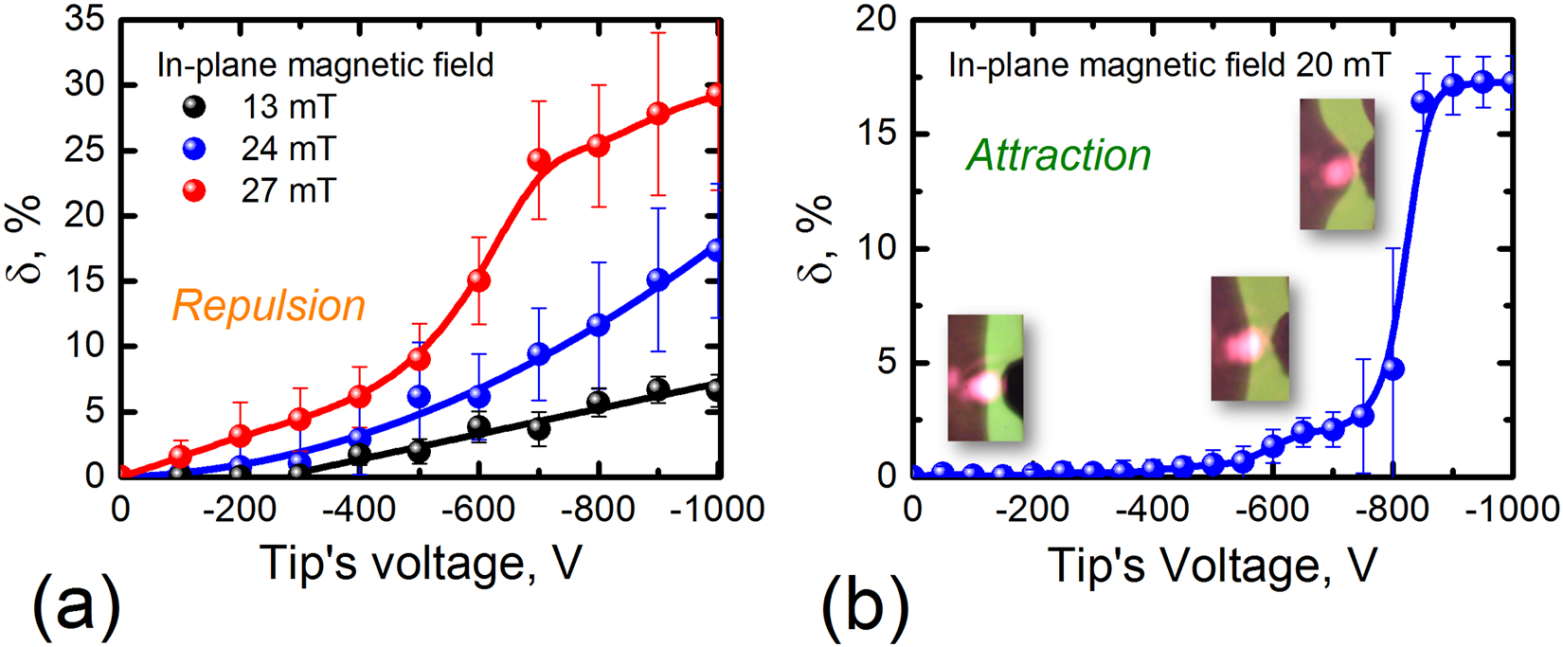
Laser intensity modulation via tip’s voltage for the “large” laser spot. The laser spot diameter is 6 μm; laser wavelength is 660 nm. Two different types of the DW-tip interaction are investigated: (**a**) repulsion and (**b**) attraction. Experimental data is shown with spheres; insets on (**b**) show the DW positions with tip’s voltage growing. In (**a**) the black line is the linear fitting of the experimental points for tip’s voltage magnitudes higher 300 V, the blue line is the quadratic fitting; other lines in (**a**) and (**b**) are Bézier splines. The in-plane magnetic fields are shown in the plots; the out-of-plain fields are 0.15, 0.3, 0.45, and 0.6 mT for the in-plane fields of 13, 20, 24, and 27 mT, respectively.

**Figure 4:**
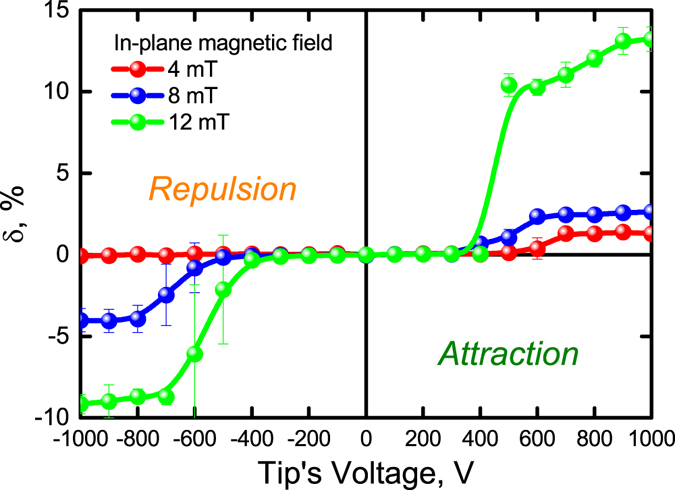
Laser intensity modulation via tip’s voltage for the “small” laser spot. The laser spot diameter is 2 μm; laser wavelength is 660 nm. Experimental data is shown with spheres; lines are Bézier splines. The in-plane magnetic fields are shown in the plot. Out-of-plane field varies from 0.03 to 0.15 mT.

Depending on the DW displacement character and laser spot size we observed different regimes of the laser modulation: linear, nonlinear and even tri-stable regimes, switching between them with in-plane magnetic field and/or sign of the tip’s voltage (Figs 3 and 4). “Large” (6-μm-diameter) and “small” (2-μm-diameter) laser spots were used to obtain smooth or abrupt intensity variations. At this, the initial DW-to-tip distance was 4 and 3 μm, respectively. The DW displacements were up to 4 μm depending on the in-plane magnetic field.

There are no sharp features in the case of DW repulsion and 6-μm-laser spot (Fig. 3a). Nevertheless, the corresponding intensity modulation character depends on the in-plane magnetic field. There is no DW shift and *δ* = 0 for small in-plane field and tip’s voltages up to 300 V; for higher voltages the modulator works in the linear regime (black spheres in Fig. 3a). Increasing the in-plane field brings higher modulation depth and nonlinearity (blue and red lines in Fig. 3a). Thus, tuning the in-plane magnetic field allows changing the response function of the modulator. On the contrary, the attraction of the DW could also show binary regime of modulation and DW displacement is step-like (Fig. 3b). The step-like modulation regimes is also possible for the DW repulsion if the “small” laser spot is used (Fig. 4). At this, smaller in-plane magnetic fields are necessary.

Large diversity of the modulation regimes is due to the DW parameters variable by the bias magnetic field: polarization **P**, chirality, and mobility^29^. In particular, increase of **P** and mobility in the in-plane magnetic field makes the DW displacement larger and might lead to nonlinear modulation (Figs 3 and 4). On the other hand, the DW chirality determines the attraction or repulsion cases (Fig. 2). In addition to these parameters, the DW coercivity is also important since it prevents DW from motion at small tip’s voltages.

The difference in the modulation character in the cases of repulsion and attraction are due to the gradient of the tip’s electric field. The magnetoelectric DW-tip interaction depends on the distance between the charged tip and bulge of DW. For the repulsion this distance increases and the magnetoelectric interaction drops. Consequently, smooth variations of intensity take place. On the contrary, for the attraction the DW-to-tip distance decreases and the magnetoelectric force becomes larger that leads to abrupt modulation of the intensity. The value of *δ* reaches maximum when DW leaves the laser spot (Fig. 3b).

## Discussion

We have proposed and demonstrated a novel concept of light control via electric field. In this scheme the magnetic domain wall plays a role of nanodevice driven by electric field. Non-uniform electric field shifts the DW in the iron garnet film due to the flexomagnetoelectric effect. Displacement of the DW modifies overall polarization rotation of the focused laser beam passing through the magnetic domains. Key advantages of the proposed approach are as follows: (i) possibility for ultra-local magnetization switching, (ii) absence of any electric current flows, and (iii) tunability of the modulation regime via constant magnetic field. The DW’s shift in the studied sample reaches 4 μm. This allows to modulate laser beam intensity in linear, nonlinear and even tri-stable regimes, switching between them with in-plane magnetic field and/or sign of the tip’s voltage. The crucial point here is that the electric field of the tip is applied locally influencing only 15–30 µm-long segment of a single DW without touching neighboring DWs. Such local control is not achievable in the conventional way (when medium magnetization is influenced by a magnetic field of a magnet) and easier in realization in comparison to ultrafast optical magnetism^11–15^.

Though here we investigate near-static magnetization switching, the magnetoelectric method of the DW control will allow operation frequencies up to 100 MHz. This is estimated from the typical DW velocity of 100 m/s in garnets. Two orders of magnitude higher operation frequency can be obtained in orthoferrites. However, this is still several times behind the electro-optical modulation rates. On the other hand, the ultrafast optical technique provides access to sub-terahertz frequencies. Consequently, the combination of all-optical and magnetioelectric techniques could open new horizons for practical applications. At the same time, the recent progress in fabrication of magnetoelectric metamaterials with high intrinsic effective magnetic fields and therefore much larger remagnetization rates^31, 32^ might significantly broaden the capabilities of the magnetoelectric devices. It allows considering the proposed method of light modulation as potentially competitive to the electro-optical one. Its main advantage is much smaller size of the modulation elements which is about several microns oppositely to the bulky electro-optical media of several centimeters in length. Other great feature of the proposed modulator is observed tri-stability which is crucial for nanophotonic logic. Deposition of plasmonic structures to enhance the magneto-optical interaction^33–36^ and further possible miniaturization of the elements down to hundreds of nanometer size makes the proposed concept of the light modulation very promising for nanophotonic and spintronic applications.

## Methods

### Experimental setup

General layout of the experiment is presented in Fig. 1. The detailed scheme of experimental setup is presented in Fig. S1, Supplementary 1. The tip made of 15-μm-diameter metallic wire is placed on a three-coordinate micrometer translation stage and the tip’s voltage is driven with a bipolar high-voltage power supply (Mantigora HV-6000 V with transistor polarity switcher). The sample is placed on translation stage between the poles of two electromagnets generating magnetic fields in-plane and normally to the sample surface. The in-plane magnetic field is required to increase the DW displacement by tuning an internal magnetic structure of the DW closer to Néel-type^20^. The out-of-plane field is applied to equalize the domains widths in the in-plane field. Diode laser with central wavelength *λ*_*0*_ = 660 nm (Thorlabs HL6545MG) is used as the light source. The laser beam passes through the system of polarizer – sample – analyzer and gets captured by silicon photodiode (Thorlabs FDS100). The light is focused on the sample in two ways: into “large” 6-µm-spot with the lens and into “small” 2-µm-spot with 10x micro-objective. White LED and USB-camera are used for checking laser focusing and its alignment with respect to the DW in crossed-polarization technique^24, 30^. The Glan-Taylor prisms with extinction ratio of 10^5^ are used as polarizer and analyzer.

### Sample

The sample is 7.4-µm thick (BiLu)_3_(FeGa)_5_O_12_ film grown on (210) gadolinium gallium garnet substrate by liquid phase epitaxy. The saturation magnetization 4*πM*_s_ = 77 G and Curie temperature 125 °C. *K*_*u*_ = 732 erg/cm^3^, *K*_*orth*_ = 5333 erg/cm^3^, *K*_*c*_ = 3208 erg/cm^3^ are constants of uniaxial, orthorhombic and cubic anisotropies, respectively.

The film demonstrates specific Faraday rotation of 0.61°/μm at wavelength of 660 nm and has a stripe domain pattern (Fig. 1, inset). In the absence of the external magnetic field the DW magnetic structure is a superposition of the Néel and Bloch type magnetization distribution. As a result, it performs relatively small flexomagnetoelectric displacement in zero magnetic biasing. It should be noted that iron garnets are nowadays considered among most promising candidates for spintronics and magnonics^28, 37, 38^.

## Electronic supplementary material

Supplemmentary 1
