# Supplementary material for: Electric-field-driven magnetic domain wall as a microscale magneto-optical shutter
Source: Sci Rep. 2017 Mar 21;7:264. doi: 10.1038/s41598-017-00365-8 (PMC5428230; doi:10.1038/s41598-017-00365-8)
Supplement: Supplementary file 1 — Supplemmentary 1 [file 41598_2017_365_MOESM1_ESM.pdf]

Electric-field-driven magnetic domain wall as a micro scale magneto-optical shutter

N.E. Khokhlov, A.E. Khramova, E.P. Nikolaeva, T.B. Kosykh, A.V. Nikolaev, A.K. Zvezdin, A.P. Pyatakov, and V.I. Belotelov

Supplementary 1

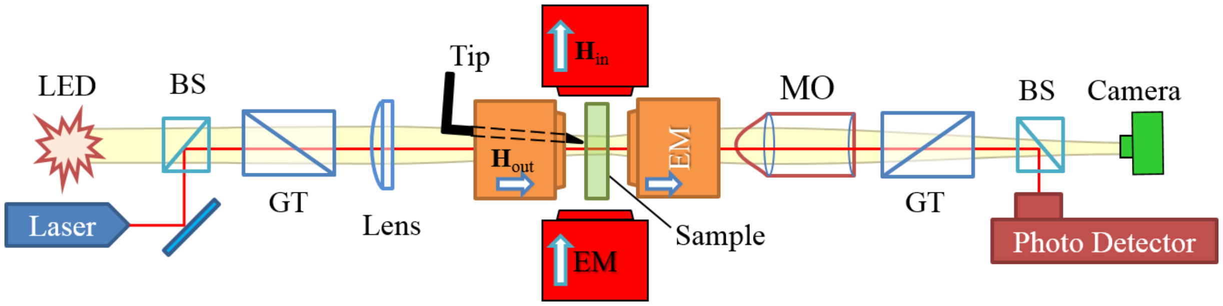

Fig.1S. Scheme of the experimental setup: BS – beam splitter, GT – Glan-Tailor prisms, EM – electromagnets, MO – micro-objective. Arrows show the direction of external magnetic fields: $\mathbf{H_{in}}$ is field in plane of the sample (in-plane field); $\mathbf{H_{out}}$ is field perpendicular to the sample surface (out-of-plane field).
